# Empowering the Sports Scientist with Artificial Intelligence in Training, Performance, and Health Management

**DOI:** 10.3390/s25010139

**Published:** 2024-12-29

**Authors:** Nuno Mateus, Eduardo Abade, Diogo Coutinho, Miguel-Ángel Gómez, Carlos Lago Peñas, Jaime Sampaio

**Affiliations:** 1 Research Center in Sports Sciences, Health Sciences and Human Development, CIDESD, CreativeLab Research Community, 5000-801 Vila Real, Portugal; eduardoabade@utad.pt (E.A.); dcoutinho@umaia.pt (D.C.); ajaime@utad.pt (J.S.); 2 Department of Sports Science, Exercise and Health, School of Life Sciences and Environment, University of Trás-os-Montes and Alto Douro, 5000-801 Vila Real, Portugal; 3 Department of Sports Sciences and Physical Education, University of Maia, 4475-690 Maia, Portugal; 4 Portugal Football School, Portuguese Football Federation, 1495-433 Oeiras, Portugal; 5 Facultad de Ciencias de la Actividad Física y del Deporte, Universidad Politécnica de Madrid, 28040 Madrid, Spain; magor_2@yahoo.es; 6 Faculty of Education and Sport Sciences, Universidad de Vigo, 36004 Vigo, Spain; arlos.lago.penhas@gmail.com

**Keywords:** data science, sports science, sensors, team sports, technology

## Abstract

Artificial Intelligence (AI) is transforming the field of sports science by providing unprecedented insights and tools that enhance training, performance, and health management. This work examines how AI is advancing the role of sports scientists, particularly in team sports environments, by improving training load management, sports performance, and player well-being. It explores key dimensions such as load optimization, injury prevention and return-to-play, sports performance, talent identification and scouting, off-training behavior, sleep quality, and menstrual cycle management. Practical examples illustrate how AI applications have significantly advanced each area and how they support and enhance the effectiveness of sports scientists. This manuscript also underscores the importance of ensuring that AI technologies are context-specific and communicated transparently. Additionally, it calls for academic institutions to update their curriculums with AI-focused education, preparing future sports professionals to fully harness its potential. Finally, the manuscript addresses future challenges, such as the unpredictable nature of team sports, emphasizing the need for interdisciplinary collaboration, including clear communication and mutual understanding between sports scientists and AI experts, and the critical balance between AI-driven insights and human expertise.

## 1. Introduction

The world of sports is undergoing a rapid digital transformation, driven by advancements in technology, particularly Artificial Intelligence (AI) [1,2,3]. This shift is revolutionizing how sports are analyzed, interpreted, and optimized across various dimensions [2,4,5,6]. AI enables computer systems to undertake tasks traditionally requiring human intelligence, such as speech recognition, decision-making, and pattern recognition, with greater efficiency. Moreover, it enables supports applications like natural language processing, video analysis, and problem-solving [1,7].

While AI has existed since the mid-20th century [8,9], recent breakthroughs, such as Generative Pretrained Transformers (GPTs), have significantly improved its ability to process vast datasets, identify patterns, and autonomously enhance system performance [6,10]. At present, AI integration in sports extends far beyond basic performance analytics, encompassing strategic planning, real-time decision-making, and exercise prescription [10]. The accessibility of AI technology, coupled with wearable trackers and physiological sensors, has led to a surge in data collection, covering performance, workload, wellness, fitness levels, and recovery [11,12,13]. This wealth of data has the potential to transform decision-making processes in sports, enabling personalized training programs, enhanced injury prevention strategies, and improved player performance.

However, the key challenge lies in converting this massive influx of raw data into actionable insights for optimizing performance [12,14]. Sports scientists now face the task of accurately analyzing these datasets to provide evidence-based feedback within strict timeframes [15]. This evolving role demands interdisciplinary knowledge, integrating sports science, data science, and machine learning into cohesive strategies. Clear communication, shared goals, and mutual respect for methodologies are essential to successful collaboration across disciplines. With the support of AI, sports scientists can achieve more precise load management, injury prevention, and performance optimization [15,16]. Nevertheless, prioritizing key performance metrics and effectively interpreting AI-generated insights remain significant challenges in this data-driven landscape.

This article explores the transformative role of AI in sports science, with a particular focus on team sports. It examines how sports scientists can effectively harness AI to optimize training loads, prevent injuries, enhance tactical insights, improve player recruitment, and manage player health. Through practical examples, this article further illustrates AI’s potential to improve sports performance, while also addressing the steps that sports scientists must take to remain at the forefront of this technological revolution.

## 2. AI in Sports Sciences

AI has emerged as having a pivotal role in reshaping the landscape of sports sciences. While sports scientists have long operated within the domains of physical activity and performance analysis, the tools now available, powered by AI, are unprecedented in their sophistication and capabilities [17]. The application of AI in sports dates to early systems such as cybernetics in the 1960s, which employed mathematical models to simulate tactical decisions in basketball. Since then, advancements have rapidly evolved, with notable milestones like the IBM’s “electronic coach” and the NBA’s “Advanced Scout”, both of which pioneered data mining for player performance analysis [18].

The use of AI terminology in sports science can frequently lead to misinterpretations. It is crucial to distinguish between different levels of AI and the tools used. Big data refers to the process of collecting and organizing vast datasets for analysis. Tools used in this context range from basic software like Excel to more complex systems like SQL and Pentaho, which are designed to filter and manage databases [19]. AI, however, takes this further by enabling intelligent decision-making based on patterns in the data. Machine learning is a branch of AI that involves algorithms which learn from data to predict outcomes or classify objects [20]. For instance, machine learning models can optimize player performance by detecting trends that influence injury risk [21]. Deep learning represents a more advanced stage of AI, employing neural networks with many layers to process complex data automatically. Deep learning excels at tasks like image and speech recognition, with models such as those predicting sports injuries becoming highly efficient [22]. Large language models, such as GPT-3, are a specific application of deep learning. These models are trained on massive datasets to generate human-like text, which could be leveraged in sports to analyze commentary, enhance fan engagement, and even create training simulations [23].

Currently, AI enables sports scientists to leverage massive datasets that were previously too complex for manual analysis, transforming data into actionable insights. These insights cover a variety of applications, including load management, injury prevention, scouting, and performance optimization. By utilizing tools such as predictive modeling and real-time analytics, AI empowers sports scientists to offer more personalized feedback for both coaches and players [14].

Wearable technology has emerged as a cornerstone of modern sports science. The data generated by these devices allows for real-time monitoring of a player’s physiological responses, providing a deeper understanding of how external and internal loads affect performance. When combined with AI, these data can refine training programs, optimize recovery protocols, and minimize the risk of overtraining and related injuries [21]. In summary, AI is revolutionizing the sports science landscape. The integration of AI technologies, from machine learning to large language models, has equipped sports scientists with tools to analyze vast datasets quickly and effectively, driving sports performance to new heights. As AI technology continues to evolve, its role in sports science will only expand, demanding that sports professionals stay abreast of the latest developments.

## 3. Load Optimization and Injury Prevention

### 3.1. Traditional Load Management Techniques

In the past, coaches heavily relied on experience and subjective assessments to manage players’ training loads. This method, though valuable, lacked precision and was time-consuming, making it particularly inefficient for the extended monitoring of performance and injury prevention [24]. The introduction of tracking systems and wearable technologies has significantly improved training load management. These systems now allow for the detailed monitoring of players’ physical activities and physiological responses, which help prevent overtraining and reduce injury risk by analyzing both external and internal loads [12,24,25,26]. However, transforming these raw data points into actionable insights remains a challenge for sports scientists. Integrating AI into data analysis allows sports scientists to provide personalized training programs, optimized injury prevention strategies, and enhanced overall performance management [14,21].

### 3.2. AI in Personalized Training and Performance Insights

Tailoring training programs to individual players remains one of the key challenges in sports. Traditional approaches often fail to accommodate the varying needs of individual players, but AI-driven tools are bridging this gap by offering real-time feedback and predictive insights. These tools leverage machine learning models, such as random forests and gradient boosting algorithms, which analyze multidimensional data, including physiological metrics, workload history, and recovery profiles, to predict optimal training loads. This enables sports scientists to make evidence-based, personalized adjustments to training programs [27,28], departing from the traditional one-size-fits-all approach. For example, systems like Athletica (Athletica Inc., Vancouver, BC, Canada) and WIMU SVIVO (Hudl, Lincoln, NE, USA) allow sports scientists to generate customized training plans using dynamic load optimization algorithms, providing immediate feedback on a player’s performance and enabling real-time adjustments to training intensity [29,30]. This precision improves the effectiveness of training sessions, maximizing each player’s strengths while minimizing potential injury risks. Moreover, AI supports long-term player development by tracking progress and identifying performance trends over time. By employing time-series analysis and unsupervised learning techniques, such as clustering algorithms, AI tools detect subtle performance trends that might otherwise go unnoticed. This allows sports scientists to gain deeper insights into how a player’s performance evolves across training cycles, further optimizing individualized training interventions [6,20].

### 3.3. AI-Driven Injury Prevention and Return to Play

Injuries are an inevitable part of sports. For instance, in men’s professional football, the incidence of hamstring muscle injuries has increased over the past decade [31]. Notably, during the 2022–2023 season, the injury costs across the top five men’s European football leagues surged by nearly 30%, rising from €553.62 million to €704.89 million [32]. This sharp increase underscores the growing challenge of injury management and the urgent need for effective prevention strategies.

In this context, AI-driven predictive analytics allow sports scientists to assess injury risks with unprecedented accuracy by employing advanced machine learning models such as decision trees, Markov processes, and neural networks [27,33,34]. These models analyze multidimensional data, such as a player’s psychological state, nutrition, sleep patterns, injury history, and genetic predispositions, to identify subtle patterns and early warning signs that traditional methods might overlook. For example, Zone7 (Zone7 Inc., Palo Alto, CA, USA) uses supervised learning techniques to predict injury risk by analyzing extensive workload data and historical injury records, offering real-time insights that help sports scientists prevent injuries and optimize player management [35]. Another example is the computer vision OpenPose (doxygen 1.9.1), which employs deep learning models and tracking algorithms to estimate human body movements in real-time 2D and 3D detection. By using techniques like the greedy bipartite matching algorithm, OpenPose provides precise and predictive information about positions and movements, allowing sports scientists to identify biomechanical inefficiencies and improve immediate corrective actions based on individual characteristics.

The role of sports scientists extends into the return-to-play phase. In this context, virtual reality and augmented reality technologies are increasingly utilized to create interactive and personalized rehabilitation programs. These systems incorporate AI-powered motion tracking and biomechanical modeling to make exercises during this stage more engaging and precise, enhancing adherence and accuracy [3,4]. Innovations such as deep reinforcement learning models, adapted from clinical settings, may further optimize rehabilitation strategies by analyzing player-specific data to tailor interventions [28].

## 4. Sports Performance Analysis and Tactical Adjustments

### 4.1. AI in Sports Performance Analysis

Sports performance analysis has evolved significantly since Charles Reep’s early work in football data collection in the 1950s, with the introduction of AI leading to dramatic advancements. One of the key developments in modern sports analysis is the integration of AI technologies that process vast datasets from wearable devices, video footage, and match statistics.

Advanced optical tracking systems, such as those employed during the FIFA World Cup 2022, utilize computer vision and deep learning algorithms to capture and analyze player and ball movements in real time [36]. These systems provide detailed metrics on players’ external load, technical and tactical performance, ball movement, and team behaviors [37]. Technologies like Second Spectrum, used in National Basketball Association (NBA) games, utilize AI and high-resolution cameras to elevate the accuracy and depth of sports analytics, offering precise analyses of player actions and game dynamics [38]. An additional example is the SkillCorner tracking system (SkillCorner, Paris, France), which delivers advanced tracking data and analytics to model and assess player performance in sports such as football, basketball, and American football.

These systems provide sports scientists and performance analysts with a comprehensive view of individual and team performance, offering actionable insights for training and competition [1,2,39]. By leveraging predictive analytics and unsupervised machine learning techniques such as clustering and dimensionality reduction, these tools also allow sports scientists to identify key contextual factors, such as opponent quality, tactical imbalances, and match congestion [40,41]. This real-time analysis is crucial for developing adaptive strategies that exploit opponents’ weaknesses. Beyond facilitating real-time analysis and strategic adjustments, these AI tools also enhance training methodologies [42,43], enabling coaches and analysts to perform scenario simulations by using reinforcement learning models that predict the outcomes of various tactical adjustments. This facilitates more dynamic decision-making, ensuring performance strategies remain flexible and data-driven during high-stakes matches.

### 4.2. AI-Driven Talent Identification and Scouting

Traditional scouting methods have been enhanced by AI’s ability to process large datasets, allowing scouting departments to evaluate a player’s potential more accurately [44]. AI-driven platforms like TwelveGPT Scout (Twelve Football, London, United Kingdom) integrate scout reports, performance metrics, and biometric data into comprehensive profiles, providing scouting departments with a more objective basis for decision-making [2,45]. For instance, AI models can simulate various scenarios to predict a player’s potential impact on team performance, allowing scouts to assess how a recruit might integrate into existing tactical frameworks [46,47]. This process gives coaches and scouts a clearer picture of how new players will contribute to the team, enhancing the efficiency of talent identification.

### 4.3. Real-Time Tactical Adjustments with AI Insights

One of the most transformative applications of AI is its ability to provide live tactical adjustments during matches. By analyzing real-time data from player tracking systems, AI tools offer suggestions for optimal strategies regarding player rotations, defensive alignments, and offensive plays [2,5,48,49]. Real-world examples, such as the Toronto Raptors’ use of a ghosting method to simulate defensive behaviors [50], and Liverpool FC’s development of the TacticAI system in collaboration with Google DeepMind, demonstrate how AI can model complex in-game scenarios to predict opponent behaviors and suggest effective counterstrategies [39]. This AI-driven feedback allows coaches to make precise adjustments that can directly impact match outcomes, particularly in high-stakes competitions where every decision counts. For example, reinforcement learning algorithms can simulate the impact of substitutions or formation changes in real time, providing coaches with data-driven recommendations to optimize team performance under dynamic conditions. As AI technology continues to evolve, its role in shaping strategic approaches will likely become even more pivotal.

## 5. Monitoring Player Health

### 5.1. Monitoring Player Health and Off-Training Behavior

Players often engage in substantial physical activity during training, but off-training periods may involve excessive sedentary behavior. Research has highlighted that players’ sedentary lifestyles during off-training periods can lead to detrimental sports and health outcomes [51,52]. Despite this, many players and coaches falsely assume that intense training is sufficient to mitigate the negative impacts of sedentary behavior. This misconception needs to be addressed to ensure optimized performance and overall health.

Intelligent wearable sensors provide advanced capabilities for monitoring and adjusting daily movement patterns outside of training [11,53]. Unlike traditional sensors that merely record data, these AI-enabled sensors incorporate technologies such as accelerometers, gyroscopes, and heart rate monitors, paired with AI algorithms, to analyze and interpret data in real time. By leveraging machine learning models and time-series analysis, these devices can assess not only physical activity but also pinpoint prolonged periods of inactivity, offering precise data on a player’s daily routines. AI-based apps like Wakeout app (Wakeout Inc., San Francisco, CA, USA), which offers quick movement breaks, and Homecourt (NEX Team Inc., San Jose, CA, USA), which gamifies skill practice, use behavioral reinforcement algorithms to personalize feedback and adapt recommendations dynamically based on user habits and responses. Additionally, AI-powered virtual assistants like Siri, Microsoft Copilot, or Google Gemini integrate natural language processing to provide continuous, personalized guidance. These systems can track user input, deliver motivational prompts, and offer real-time adjustments to activity goals, helping players maintain healthy behaviors and avoid health declines. Research indicates that players who actively monitor their daily movement outside of training can reduce their risk of sedentary lifestyle-related issues, such as decreased cardiovascular fitness and increased injury rates [54].

### 5.2. AI-Powered Sleep Quality Enhancement

Adequate sleep is crucial for elite players, directly impacting energy recovery, cognitive function, and overall performance [55,56]. However, players often face challenges in achieving the necessary sleep duration and quality due to intensive travel schedules, late-night games, and lifestyle factors [57,58]. Poor sleep not only hampers physical performance but also increases the risk of injuries and cognitive impairments, such as slower decision-making and reduced tactical accuracy [55,56,59,60].

AI technologies significantly enhance the monitoring and optimization of sleep patterns by leveraging wearable devices equipped with advanced biosensors. These wearables, such as WHOOP (WHOOP Inc., Boston, MA, USA), track critical sleep-related biometrics such as heart rate variability and sleep cycles. These tools provide personalized recommendations for optimizing sleep and managing recovery. By analyzing extensive sleep data, AI can detect subtle patterns and disturbances, offering individualized strategies to improve both short-term recovery and long-term well-being [10].

### 5.3. Menstrual Cycle Management with AI

Managing the menstrual cycle is an often-overlooked aspect of optimizing female players’ performance. Although research on the menstrual cycle’s effects on performance parameters has produced mixed results [61,62], many female athletes report that their cycle affects both training and performance [63,64]. Tailoring training programs to different phases of the cycle can improve performance and alleviate discomfort [65]. For example, female players may face increased training strain and extended recovery periods during specific phases, highlighting the need for adjusted training intensities and recovery strategies [66,67].

AI offers innovative solutions for menstrual cycle management by predicting ovulation dates and identifying risks linked to conditions such as Premenstrual Syndrome (PMS) or Luteal Phase Defect through the analysis of menstrual data [68]. Furthermore, AI-driven tools can accurately classify menstrual phases using biometric data such as heart rate or ECG signals, with accuracy rates exceeding 85% [69]. These predictive capacities enable sports scientists to craft more precise and personalized training plans that align with a player’s hormonal fluctuations, improving both physical outcomes and mental well-being.

## 6. Ethical Considerations in AI Integration in Sports Sciences

As AI becomes more integrated into sports science, several ethical concerns and challenges arise, particularly related to data privacy and the potential for over-reliance on technology. AI systems in sports often collect vast amounts of sensitive information, including biometric data, physiological metrics, and personal details, raising concerns about how these data are stored, shared, and used [11]. To mitigate these risks, sports scientists must ensure that AI applications comply with data protection laws, like the General Data Protection Regulation (GDPR) in Europe or other privacy frameworks [70]. Transparent communication with players about data collection practices and their benefits is crucial to maintaining trust and ensuring ethical practices. Another concern is the possible over-reliance on AI, which could diminish the role of human intuition and expertise in decision-making. While AI tools provide valuable insights, they should complement human judgment rather than replace it, particularly when dealing with the unpredictable nature of sports.

## 7. Integrating AI-Focused Education in Sports Science Curriculums

The rise of AI in sports science demands that academic institutions adapt their curricula to equip the next generation of sports professionals with the necessary skills. This includes providing comprehensive training in data analysis, machine learning, and the ethical implications of AI technologies [71]. Future sports scientists must be adept at interpreting complex AI-generated data and transforming these insights into practical training and recovery strategies. Additionally, students should learn data visualization techniques and the use of dynamic interfaces to present complex data in a user-friendly format. By doing so, they will be better equipped to collaborate with coaches and decision-makers, ensuring that AI insights are actionable and easily interpretable.

## 8. Future Challenges and Opportunities

While AI offers tremendous benefits to sports science, fundamentally changing how players train, perform, and manage their well-being, challenges remain. One of the key obstacles is the unpredictability of sports, where AI models may struggle to account for human physiological variability and complex team dynamics. AI systems must continue to evolve to handle these complexities, combining large datasets with improved algorithms that can better predict outcomes in highly variable conditions.

Interdisciplinary collaboration between sports scientists, data scientists, and AI engineers is essential to developing AI tools tailored to the unique needs of sports. Successful collaboration demands clear communication, shared goals, and mutual respect for each discipline’s methods. Sports scientists must grasp AI fundamentals to interpret outputs effectively, while AI engineers must understand the complexities of sports data, such as variability in player performance and contextual factors. Without such collaboration, AI applications risk becoming technically sound but impractical or oversimplified. Establishing robust frameworks for interdisciplinary teamwork is critical to ensure that AI innovations are both scientifically robust and practically relevant.

Another significant challenge is the limited availability of open-source, peer-reviewed scientific validation for many of the sensor technologies and AI applications currently in use. While these tools are increasingly adopted in professional sports and show significant promise, comprehensive validation studies confirming their reliability and accuracy are often limited or absent. This gap highlights the need for future research to systematically evaluate these technologies under controlled conditions to ensure their effectiveness and generalizability. Addressing this limitation is critical for fostering trust among stakeholders and integrating these tools more seamlessly into evidence-based practices.

Despite the many advantages of AI, it is unlikely to replace human expertise in sports science. Human intuition, experience, and the ability to read situational context remain invaluable. While AI systems can process vast amounts of data, they often struggle with the unpredictable nature of sports, such as unexpected injuries, changes in team dynamics, or psychological factors affecting performance. Therefore, sports scientists must strike a balance between relying on AI for data-driven insights and applying their expertise to make informed decisions. Viewing AI as a tool that augments human capabilities, rather than replaces them, allows sports professionals to develop more holistic and adaptable strategies.

## 9. Conclusions

**Figure 1:**
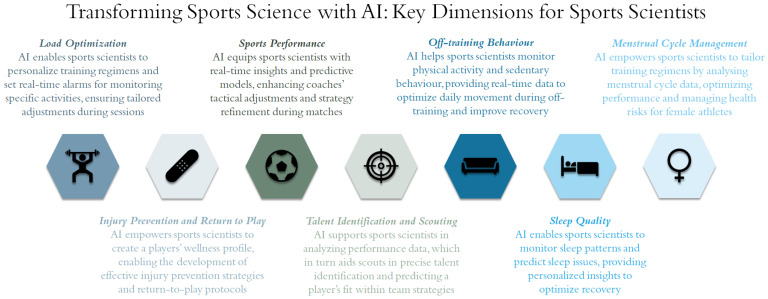
Diagram illustrating key AI-driven dimensions for sports scientists.

AI is transforming sports science, empowering sports scientists with innovative tools to optimize training, enhance performance, and manage player health. While AI cannot fully replace human expertise, it acts as a complementary asset that enhances the decision-making of sports scientists, ultimately improving player outcomes. To unlock AI’s full potential, it is essential to employ it responsibly, ensuring that it supports human judgment rather than undermines it. By harnessing AI across the key dimensions of sports science, as illustrated in Figure 1, sports scientists can gain deeper insights into player and team performance.

However, the integration of AI also presents challenges. Ethical considerations, particularly concerning data privacy, must be carefully addressed. AI should amplify human intuition and expertise rather than automate decisions. As highlighted in Table 1, various practical applications demonstrate how AI can boost evidence-based decision-making, especially in areas such as load optimization, injury prevention and return-to-play, sports performance analysis, talent identification and scouting, off-training behavior, sleep quality enhancement, and menstrual cycle management.

Considering these advancements, academic institutions and sports organizations must evolve their educational programs to equip future sports professionals with the necessary AI skills to navigate and excel in this rapidly advancing field.

## Figures and Tables

**Table 1:** AI applications in sports science: key dimensions and examples involving sports scientists.

Dimension	Hypothetical Scenario	Practical Example
Load Optimization	The coaching staff of a professional football team needs to tailor each player’s training load according to their individual physical profile and recovery requirements. This approach aims to optimize performance throughout the training cycle.	An AI system tracks a central midfielder’s sprint and acceleration profiles in real time. When the sports scientist receives an alert from the system that the player is approaching his workload limit, it notifies the coaches. The training program may then be adjusted by decreasing the volume of high-intensity activities, effectively managing the player’s load.
Injury Prevention and Return to Play	The coaching staff of a professional basketball team need to design a plan for a player’s return-to-play process. Their aim is to ensure a safe and effective reintegration into training and competition while minimizing the risk of re-injury.	A basketball point guard recovering from a hamstring injury had a comprehensive profile established before the injury using AI tools. This pre-injury data support his return-to-play strategy, with the AI system continuously monitoring his performance metrics and comparing them to his baseline (i.e., pre-injury). Sports scientists use these insights to guide the coaching staff in adjusting the player’s training regimen.
Sports Performance	A basketball team is underperforming during a game, and the coaching staff needs to quickly understand and address the issues. The aim is to use AI to analyze real-time data and historical performance to make immediate tactical adjustments and improve team performance.	AI identifies that a key player is shooting poorly and provides insights into the opponent’s defensive patterns. The system recommends the best player to substitute in, based on their current and historical performance metrics. Additionally, the coach may use the AI’s insights to adjust the offensive strategy and team dynamics, targeting the opponent’s weaknesses more effectively.
Talent Identification and Scouting	A football team needs to replace a forward who is leaving the club. The challenge is to use AI to identify and evaluate potential new forwards.	An AI system analyzes performance metrics, playing style, and biometric data of potential players and compares them with those of the departing forward. The system identifies a candidate who closely matches the departing player’s profile. Sports scientists then collaborate with scouts to conduct further evaluations and ensure the candidate’s suitability aligns with the team’s strategy and needs.
Off-training Behavior	A rugby player exhibits abnormal recovery metrics (e.g., objective and/or subjective) despite a standard training profile, potentially due to insufficient off-training physical activity or prolonged sedentary periods.	AI monitors the player’s daily movement patterns and delivers real-time feedback to adjust off-training activity recommendations. Sports scientists use these insights, combined with AI-powered virtual assistants that provide continuous guidance and motivation, to ensure the player maintains healthy behaviors. Ultimately, this approach might optimize recovery.
Sleep Quality	A professional basketball player is experiencing sleep disturbances due to frequent travel and late-night games. These issues are affecting the player’s recovery and performance.	Sports scientists employ advanced wearable devices to monitor the player’s sleep patterns and disruptions linked to travel and night games. AI analyzes the collected data to pinpoint sleep issues and generate actionable insights. Based on this information, tailored sleep strategies are devised, including adjustments to travel schedules and personalized sleep recommendations. AI-powered tools then offer real-time feedback and guidance, helping the player optimize sleep quality.
Menstrual Cycle Management	Female players of a football team report variations in their physical performance related to different phases of their menstrual cycle. Sports scientists also detect fluctuations in training strain and intensity throughout these phases.	AI helps to understand historical performance variations across different menstrual phases, providing insights into how performance fluctuates. Based on these insights, sports scientists can suggest adjustments in training programs to align with the player’s cycle phase, aiming to optimize overall performance.
